# Frequency and Types of Patient-Reported Errors in Electronic Health Record Ambulatory Care Notes

**DOI:** 10.1001/jamanetworkopen.2020.5867

**Published:** 2020-06-09

**Authors:** Sigall K. Bell, Tom Delbanco, Joann G. Elmore, Patricia S. Fitzgerald, Alan Fossa, Kendall Harcourt, Suzanne G. Leveille, Thomas H. Payne, Rebecca A. Stametz, Jan Walker, Catherine M. DesRoches

**Affiliations:** 1 Department of Medicine, Beth Israel Deaconess Medical Center, Boston, Massachusetts; 2 Harvard Medical School, Boston, Massachusetts; 3 Department of Medicine, David Geffen School of Medicine, University of California, Los Angeles; 4 Department of Epidemiology, University of Michigan, Ann Arbor; 5 Department of Nursing, College of Nursing and Health Sciences, University of Massachusetts, Boston; 6 Department of Medicine, University of Washington School of Medicine, Seattle; 7 Steele Institute for Health Innovation, Geisinger, Danville, Pennsylvania

## Abstract

**Importance:**

As health information transparency increases, patients more often seek their health data. More than 44 million patients in the US can now readily access their ambulatory visit notes online, and the practice is increasing abroad. Few studies have assessed documentation errors that patients identify in their notes and how these may inform patient engagement and safety strategies.

**Objective:**

To assess the frequency and types of errors identified by patients who read open ambulatory visit notes.

**Design, Setting, and Participants:**

In this survey study, a total of 136 815 patients at 3 US health care organizations with open notes, including 79 academic and community ambulatory care practices, received invitations to an online survey from June 5 to October 20, 2017. Patients who had at least 1 ambulatory note and had logged onto the portal at least once in the past 12 months were included. Data analysis was performed from July 3, 2018, to April 27, 2020.

**Exposures:**

Access to ambulatory care open notes through patient portals for up to 7 years (2010-2017).

**Main Outcomes and Measures:**

Proportion of patients reporting a mistake and how serious they perceived the mistake to be, factors associated with finding errors characterized by patients as serious, and categories of patient-reported errors.

**Results:**

Of 136 815 patients who received survey invitations, 29 656 (21.7%) responded and 22 889 patients (mean [SD] age, 55.16 [15.96] years; 14 447 [63.1%] female; 18 301 [80.0%] white) read 1 or more notes in the past 12 months and completed error questions. Of these patients, 4830 (21.1%) reported a perceived mistake and 2043 (42.3%) reported that the mistake was serious (somewhat serious: 1563 [32.4%]; very serious: 480 [9.9%]). In multivariable analysis, female patients (relative risk [RR], 1.79; 95% CI, 1.72-1.85), more educated patients (RR, 1.38; 95% CI, 1.29-1.48), sicker patients (RR, 1.89; 95% CI, 1.84-1.94), those aged 45 to 64 years (RR, 2.23; 95% CI, 2.06-2.42), those 65 years or older (RR, 2.00; 95% CI, 1.73-2.32), and those who read more than 1 note (2-3 notes: RR, 1.82; 95% CI, 1.34-2.47; ≥4 notes: RR, 3.09; 95% CI, 2.02-4.73) were more likely to report a mistake that they found to be serious compared with their reference groups. After categorization of patient-reported very serious mistakes, those specifically mentioning the word *diagnosis* or describing a specific error in current or past diagnoses were most common (98 of 356 [27.5%]), followed by inaccurate medical history (85 of 356 [23.9%]), medications or allergies (50 of 356 [14.0%]), and tests, procedures, or results (30 of 356 [8.4%]). A total of 23 (6.5%) reflected notes reportedly written on the wrong patient. Of 433 very serious errors, 255 (58.9%) included at least 1 perceived error potentially associated with the diagnostic process (eg, history, physical examination, tests, referrals, and communication).

**Conclusions and Relevance:**

In this study, patients who read ambulatory notes online perceived mistakes, a substantial proportion of which they found to be serious. Older and sicker patients were twice as likely to report a serious error compared with younger and healthier patients, indicating important safety and quality implications. Sharing notes with patients may help engage them to improve record accuracy and health care safety together with practitioners.

## Key Points

**Question:**

How often do patients who read open ambulatory visit notes perceive mistakes, and what types of mistakes do they report?

**Findings:**

In this survey study of 136 815 patients, 29 656 provided a response, and 1 in 5 patients who read a note reported finding a mistake and 40% perceived the mistake as serious. Among patient-reported very serious errors, the most common characterizations were mistakes in diagnoses, medical history, medications, physical examination, test results, notes on the wrong patient, and sidedness.

**Meaning:**

As health information transparency increases, patients may perceive important errors in their visit notes, and inviting them to report mistakes that they believe are very serious may be associated with improved record accuracy and patient engagement in safety.

## Teaser

This survey study assesses the frequency and types of errors identified by patients who read open ambulatory visit notes.

## Introduction

Errors in electronic health records (EHRs) are common.^1,2^ At least half of EHRs may contain an error, many related to medications.^2,3,4,5,6,7,8^ Overburdened practitioners may import inaccurate medication lists, propagate other erroneous information electronically by copying and pasting older parts of the record, or enter erroneous examination findings.^2,8,9^ EHRs may also lack critical information (errors of omission) because of limited interoperability among health care sites.^10^ Among primary care physicians sharing notes with patients, 26% anticipated that patients would find nontrivial errors.^11^ Despite these known problems, systems for checking the accuracy of notes are almost nonexistent. As practitioners integrate EHR data into decision-making, such errors could therefore lead to medication errors, wasteful duplication, unnecessary or incorrect treatment, and delayed diagnoses.^7,12,13,14,15^

Cultural shifts toward transparency along with nationwide growth of secure electronic patient portals may be associated with increases in patients accessing their medical records, including visit notes (open notes). Beginning in 2010, with 20 000 patients participating in a demonstration research project, open notes are now available to more than 44 million patients in 200 health care centers in every state in the US, and this practice is extending globally (eAppendix in the Supplement).^16,17^ Because of the 21st Century Cures Act to ensure patient access to electronic health information along with subsequent actions by the Office of the National Coordinator for Health Information Technology addressing patients’ legal right to access their notes through patient portals or third-party apps, this number is likely to increase.

Reading open notes may be associated with enhanced patient engagement and with improved patient safety and care quality.^11,17,18,19,20,21,22,23,24^ Patients reported that they understand notes and that reading notes helps them remember next steps (such as tests and referrals).^18,21^ They also reported that note reading enables timely follow-up of results and supports family or friend care partners with information.^18,20,21,25,26^ In addition, sharing notes with patients creates a new mechanism for patients to identify documentation errors.^27,28^

Patients and families hold unique knowledge about themselves and their care, and their reports have potential for improving individual and organizational safety.^29,30,31,32,33,34^ They experience aspects of care not seen by practitioners, such as events that transpire between visits or care transitions.^18,29,35,36^ Patients and families may also detect breakdowns in care, including some events missed by practitioners,^18,29,37,38,39,40,41,42,43,44^ and aggregate patient reports can identify organizational strengths and weaknesses.^29^ We aimed to assess the proportion of patients who perceive mistakes in ambulatory notes and how serious they perceive the mistakes to be, patient factors associated with finding mistakes that they considered somewhat or very serious, and the types of mistakes patients describe as very serious.

## Methods

### Participants

For this study, from June 5 to October 20, 2017, we surveyed patients in 3 health systems sharing notes with patients for up to 7 years^17^: Beth Israel Deaconess Medical Center, an academic medical center in Massachusetts and 6 affiliated sites; Geisinger, an integrated health system in rural Pennsylvania and southern New Jersey, including 7 hospitals and 53 community practices; and University of Washington Medicine, an urban health system in Washington with 3 hospitals and 9 freestanding practices.^23^ Each of these organizations participated in the initial open notes study in 2010. By 2014, they had expanded open notes access to almost every ambulatory medical and surgical practice and to all types of practitioners (eAppendix in the Supplement).^23^ Data analysis was performed from July 3, 2018, to April 27, 2020. The institutional review boards at Beth Israel Deaconess Medical Center, University of Washington, and Geisinger approved the study protocols at their sites. Completing the survey was voluntary, and all answers were confidential; therefore, the institutional review boards determined that informed consent was implied by survey participation. This study followed the American Association for Public Opinion Research (AAPOR) reporting guideline.

Participants were 18 years or older, had at least 1 ambulatory note, and had logged onto the portal at least once in the past 12 months. We did not include patients who had been invited to participate in other open notes research projects in the prior 12 months or who had previously opted out of all research, based on organizational limits on recruiting patients.

### Survey

The survey was adapted from the original OpenNotes questionnaire.^17^ To help focus patients on their notes, the survey included a screenshot of the location of notes on each organization’s patient portal. The survey included 4 questions about mistakes: “How confident do you feel in your ability to find mistakes in your visit notes?” (4-point ordinal responses: not at all to very confident) and “Have you ever found anything in your visit notes you thought was a mistake (not counting misspellings or typographical errors)?” (responses: no, yes, or don’t know/not sure). Those who answered yes were asked, “How important was the most serious mistake you found?” (responses: not at all serious, somewhat serious, or very serious). At 2 sites, those who described the mistake as somewhat or very serious were asked, “Please describe the most serious mistake” (free text). Respondents identified themselves as Spanish, Hispanic, Latino ethnicity (categorized as Hispanic) or not and as white, black or African American (categorized as black), American Indian or Pacific Native, Asian, Native Hawaiian or Pacific Islander, or other. Additional survey details have been published, and the questionnaire is available on request.^23,24^

### Statistical Analysis

We defined note readers as those with concordant self-reported note reading and portal tracking data. We used administrative data to compare respondent and nonrespondent characteristics. We used descriptive statistics for showing the number and proportions of note readers who reported mistakes in their notes and how important they thought the mistakes were. In this article, the terms *mistake* and *error* are used interchangeably, consonant with the variability of the terms used in the literature. All patient-reported errors reflect perceived errors.

We examined respondents’ self-reported sociodemographic and health characteristics and note reading experiences both overall and according to whether they found a mistake. For reading experience, we examined both duration (weeks, months, or years) and number of notes read (1 to ≥4). We calculated frequencies and proportions for all categorical and ordinal variables.

We modeled the probability that a patient found a somewhat serious or very serious mistake using a generalized estimating equation with a log link and binomial error.^45^ Independent variables in our model were race, ethnicity, self-reported health, educational attainment, age, sex, number of notes read, length of time reading notes, and primary language spoken at home, reflecting the hypotheses that white, more educated, and sicker patients and those who read more notes or speak English primarily at home would be more likely to report a mistake.

We used a working exchangeable correlation structure^45^ (specifying that all observations within a given cluster have equal variance and are equally correlated within each cluster) in our model to account for clustering within study site and to calculate the relative risks (RRs) and 95% CIs and checked for collinearity across covariates. All analyses were conducted using SAS statistical software, version 9.4 (SAS Institute Inc).

Drawing on a classification system previously used to analyze patient-reported note errors,^18^ we analyzed a subset of all free-text descriptions, focusing on the mistakes reported by patients as very serious. We reviewed all patient responses and added codes to the classification system to reflect new emerging categories in the data^46^ and defined each code in detail (eTable in the Supplement). Three coders (including S.K.B., K.H.) then independently reviewed a subset of the data, applying the revised codebook, with further category refinement after consensus discussion. One physician (S.K.B.) with experience in patient-reported errors then applied the final codebook to the entire data set, assigning up to 2 mistake types for each free-text description. We excluded comments that were irrelevant or indecipherable. In cases in which the mistake type was not immediately evident, we used an adjudication process with a second reviewer (C.M.D). Both reviewers discussed the text until they reached consensus on the assigned categories. Coding was done in REDCap (Research Electronic Data Capture).

## Results

### Respondents and Patient-Reported Mistakes

Of 136 815 patients who received survey invitations, 29 656 (21.7%) responded and 22 889 patients (mean [SD] age, 55.16 [15.96] years; 14 447 [63.1%] female; 18 301 [80.0%] white) read 1 or more notes in the past 12 months and completed error questions, comprising the study population (Figure). A total of 16 810 patients (73.4%) reported reading notes for at least 1 year, and 11 391 (49.8%) reported reading 4 or more notes. Among all patients, 18 437 (80.5%) reported that they were confident or very confident in their ability to find mistakes whether or not they reported a mistake in their notes. In total, 4830 of 22 889 note readers (21.1%) perceived a mistake in their notes. Univariate associations between patient characteristics and reporting a perceived error are given in Table 1.

**Figure.**
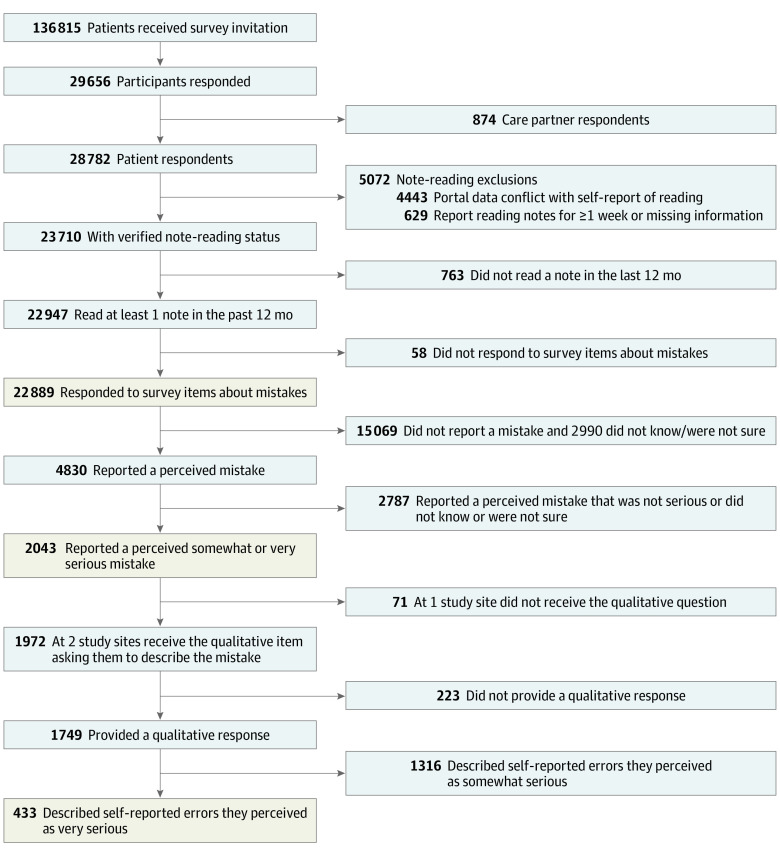
Study Participant Flowchart

**Table 1.** Characteristics of Patients Who Read 1 or More Notes in the Past 12 Months by Whether They Reported Finding a Mistake

Characteristic	Patients, No. (%)			
	Total (N = 22 889)	Found a mistake in the notes		
		Yes (n = 4830)	No (n = 15 069)	Do not know or not sure (n = 2990)
Age group, y				
18-24	774 (3.4)	87 (11.2)	568 (73.4)	119 (15.4)
25-44	5090 (22.2)	910 (17.9)	3469 (68.2)	711 (14.0)
45-64	9494 (41.5)	2114 (22.3)	6174 (65.0)	1206 (12.7)
≥65	7531 (32.9)	1719 (22.8)	4858 (64.5)	954 (12.7)
Sex				
Female	14 447 (63.1)	3426 (23.7)	9112 (63.1)	1909 (13.2)
Male	8442 (36.9)	1404 (16.6)	5957 (70.6)	1081 (12.8)
Race				
Asian	1175 (5.1)	141 (12)	851 (72.4)	183 (15.6)
Black	570 (2.5)	92 (16.1)	398 (69.8)	80 (14.0)
White	18 301 (80.0)	3941 (21.5)	12 155 (66.4)	2205 (12.0)
Other	729 (3.2)	161 (22.1)	454 (62.3)	114 (15.6)
Multiple races	786 (3.4)	187 (23.8)	474 (60.3)	125 (15.9)
Unknown	1328 (5.8)	308 (23.2)	737 (55.5)	283 (21.3)
Ethnicity				
Hispanic/Latino	816 (3.6)	149 (18.3)	559 (68.5)	108 (13.2)
Non-Hispanic	20 855 (91.1)	4398 (21.1)	13 822 (66.3)	2635 (12.6)
Unknown	1218 (5.3)	283 (23.2)	688 (56.5)	247 (20.3)
Language spoken at home				
English	19 966 (87.2)	4292 (21.5)	13 192 (66.1)	2482 (12.4)
Spanish	75 (0.3)	10 (13.3)	56 (74.7)	9 (12.0)
Other	448 (2.0)	51 (11.4)	335 (74.8)	62 (13.8)
Multiple languages	1223 (5.3)	213 (17.4)	821 (67.1)	189 (15.5)
Unknown	1177 (5.1)	264 (22.4)	665 (56.5)	248 (21.1)
Educational level				
High school or less	1456 (6.4)	175 (12.0)	1061 (72.9)	220 (15.1)
Some college or technical school	4814 (21.0)	845 (17.6)	3272 (68)	697 (14.5)
4-y College degree or some graduate school	7464 (32.6)	1509 (20.2)	4977 (66.7)	978 (13.1)
Master’s or doctoral degree	8145 (35.6)	2085 (25.6)	5184 (63.6)	876 (10.8)
Unknown	1010 (4.4)	216 (21.4)	575 (56.9)	219 (21.7)
General health				
Excellent, very good, or good	18 437 (80.5)	3665 (19.9)	12 514 (67.9)	2258 (12.2)
Fair or poor	3388 (14.8)	940 (27.7)	1941 (57.3)	507 (15)
Unknown	1064 (4.6)	225 (21.1)	614 (57.7)	225 (21.1)
Employment				
Employed or self-employed	12491 (54.6)	2427 (19.4)	8538 (68.4)	1526 (12.2)
Homemaker, unemployed, and disabled	2489 (10.9)	619 (24.9)	1466 (58.9)	404 (16.2)
Retired	6828 (29.8)	1548 (22.7)	4447 (65.1)	833 (12.2)
Unknown	1081 (4.7)	236 (21.8)	618 (57.2)	227 (21)
Works in health care				
Yes	3204 (14)	990 (30.9)	1897 (59.2)	317 (9.9)
No	18 571 (81.1)	3610 (19.4)	12 524 (67.4)	2437 (13.1)
Unknown	1114 (4.9)	230 (20.6)	648 (58.2)	236 (21.2)
Site				
BIDMC	11 911 (52)	2857 (24)	7609 (63.9)	1445 (12.1)
Geisinger	1206 (5.3)	138 (11.4)	859 (71.2)	209 (17.3)
UW	9772 (42.7)	1835 (18.8)	6601 (67.6)	1336 (13.7)
No. of notes read				
1	1691 (7.4)	168 (9.9)	1266 (74.9)	257 (15.2)
2 or 3	8836 (38.6)	1424 (16.1)	6245 (70.7)	1167 (13.2)
≥4	11 391 (49.8)	3078 (27)	7009 (61.5)	1304 (11.4)
Do not know or not sure	971 (4.2)	160 (16.5)	549 (56.5)	262 (27)
Length of time reading notes				
≤1 wk	514 (2.2)	69 (13.4)	368 (71.6)	77 (15)
>1 wk to <1 y	5565 (24.3)	823 (14.8)	3897 (70)	845 (15.2)
≥1 y	16 810 (73.4)	3938 (23.4)	10 804 (64.3)	2068 (12.3)

Abbreviations: BIDMC, Beth Israel Deaconess Medical Center; UW, University of Washington.

### Characteristics of Patients Who Found Somewhat Serious or Very Serious Mistakes in Notes

Among 4830 patients who perceived mistakes in notes, 2043 (42.3%) described them as serious (1563 [32.4%] were somewhat serious and 480 [9.9%] were very serious). In multivariable analysis, female patients (RR, 1.79; 95% CI, 1.72-1.85), more educated patients (RR, 1.38; 95% CI, 1.29-1.48), sicker patients (RR, 1.89; 95% CI, 1.84-1.94), those aged 45 to 64 years (RR, 2.23; 95% CI, 2.06-2.42), those 65 years or older (RR, 2.00; 95% CI, 1.73-2.32), and those who read more than 1 note (2-3 notes: RR, 1.82; 95% CI, 1.34-2.47; ≥4 notes: RR, 3.09; 95% CI, 2.02-4.73) were more likely to report a serious mistake compared with their reference groups (Table 2).

**Table 2.** Multivariable Analysis of Patient Characteristics and Likelihood of Reporting a Somewhat Serious or Very Serious Mistake in Visit Notes^a^

Characteristic	Relative Risk (95% CI)
Age group y	
18-24	1 [Reference]
25-44	1.81 (1.6-2.05)
45-64	2.23 (2.06-2.42)
≥65	2.00 (1.73-2.32)
Sex	
Male	1 [Reference]
Female	1.79 (1.72-1.85)
Race	
White	1 [Reference]
Black	0.97 (0.77-1.2)
Asian	0.63 (0.57-0.7)
Other or multiple races	1.28 (1.19-1.37)
Ethnicity	
Non-Hispanic	1 [Reference]
Hispanic or Latino	0.93 (0.58-1.49)
Primary language spoken at home	
English	1 [Reference]
Multiple languages	0.86 (0.85-0.87)
Other vs English	0.60 (0.57-0.63)
Spanish vs English	0.98 (0.37-2.6)
Educational level	
4-y College degree or some graduate school	1 [Reference]
High school or less	0.57 (0.44-0.74)
Some college or technical school	0.81 (0.76-0.87)
Master’s or doctoral degree	1.38 (1.29-1.48)
General health	
Excellent, very good, or good	1 [Reference]
Fair or poor	1.89 (1.84-1.94)
No. of notes read in previous 12 mo	
1	1 [Reference]
2 or 3	1.82 (1.34-2.47)
≥4	3.09 (2.02-4.73)
Length of time reading notes	
≤1 wk	1 [Reference]
>1 wk to <1 y	0.76 (0.51-1.14)
≥1 y	0.98 (0.75-1.28)

^a^ Generalized estimating equation model with working exchangeable correlation structure with study site as repeated subject.

Asian patients were less likely than white patients to report finding a serious mistake (RR, 0.63; 95% CI, 0.57-0.70), but no significant differences were found between patients identifying as black vs white or between Hispanic vs non-Hispanic patients. Patients reporting multiple races were more likely to report serious errors (RR, 1.28; 95% CI, 1.19-1.37). Patients who reported speaking multiple languages (RR, 0.86; 95% CI, 0.85-0.87) or a language other than English or Spanish as their primary language at home (RR, 0.6; 95% CI, 0.57-0.63) were less likely to report a serious error, but no meaningful differences were found between patients who primarily spoke English or Spanish at home.

### Categories of Mistakes Described by Patients as Very Serious

Among 480 patients who reported finding a very serious mistake, 463 (96.5%) were respondents in the 2 organizations where patients were asked to describe the event, and 433 (93.5%) provided a free-text description. Seventy-seven comments did not contain enough information to be classified (ie, “It was already discussed with the provider”) and were excluded.

Of the remaining 356 patient reports, the most common category of mistakes reported were those specifically mentioning the word *diagnosis* or describing a perceived error in current or past diagnoses (98 of 356 [27.5%]) (Table 3). Other very serious patient-reported mistakes included inaccurate description of medical history (85 of 356 [23.9%]); medications or allergies (50 of 356 [14.0%]); tests, procedures, or results (30 of 356 [8.4%]); and perceived errors pertaining to the physical examination, including elements of the examination that, according to the patient, were documented but not done (24 of 356 [6.7%]). In addition, 24 patients (6.7%) described failed communication (issues that the practitioner documented as said or done but that the patient perceived did not happen at the visit), such as informed consent or counseling on specific topics. A total of 23 patients (6.5%) reported reading notes on the wrong patient, and 12 (3.4%) detected errors in sidedness (left vs right).

**Table 3.** Categories and Examples of Very Serious Mistakes Reported by Patients

Category	Mistakes, No. (%) (n = 356)^a^	Example of patient-reported mistake
*Diagnosis* mentioned	98 (27.5)	“The note said I was not BRCA1 when I am BRCA1…My provider, who was surprised, immediately corrected it.” “The diagnosis for the visit was incorrect. Although I did receive a referral for physical therapy, it was for the wrong body part. This necessitated two more visits to the clinic...and a significant delay in the treatment of my injury.” “It was stated in notes that I had lung cancer. I do not and never have had lung cancer. I did have a spontaneous pneumothorax in 1976, however.”
Medical history	85 (23.9)	“Doctor reported that I did not claim to have pain in my hand. I am a pianist and I went specifically because pain was in my hand.” “I have been complaining of difficulty breathing [for over 3 mo]. It has been a real and increasing problem for me, but is not mentioned in my notes...in fact, notes saying my breathing is normal, are made for each visit.”
Medications or allergies	50 (14.0)	“Not listing my allergy that I have had anaphylaxis with before, prior to being in the operating room.” “I found an incorrect dosage on a note that was being used by a referred physician for an infusion. The dosage was incorrect by 10-fold.”
Tests, procedures, or results	30 (8.4)	“Pathology report summary stated I had 2 positive lymph nodes but detailed report stated 3 positive lymph nodes. That changes the staging and the treatment options. My physician had only read the summary and didn't realize I had 3 positive lymph nodes.” “Was counseled to have an elective abortion for blighted ovum because the sac had reached 25 mm without fetal pole or yolk sac being visible on ultrasound. [I] compared [the] note to my ultrasound report [which] noted it was only 2.5 mm.”
Physical examination	24 (6.7)	“The notes reflect exams not done in the exam room. As a medical profession I find this alarming” “Wrong BMI; told [as a result that] I was not to be put on a heart lung transplant list.”
Social history	24 (6.7)	“Although I have 6 y sober, per my medical record I drink socially...I DONT DRINK! but when a new Dr reads my record they [think I] do...Patients should be able to write the same note about their appointment, with the 2 notes together you would get a REAL idea of what happened.” “The mistake was [not] changing the language in my history to recovery instead of active drug user and the drug I was using. I had gotten into recovery and it bothered me greatly that my history kept saying I used drugs.”
Failed communication: did not happen at visit	24 (6.7)	“A provider not only failed to mention risks and side effects of the injection he wanted to give, but sarcastically derided my concern about long-term risks: 'Where'd you hear that, the Internet?' ...The 'mistake' was the lie that he discussed the risks and benefits of the procedure, when in fact all he said was that he'd done hundreds of such injections.” “The surgeon...mentioned that I had taken a decision not to have further tissue removed. But I did not do such thing. Actually I asked her why she did not follow the golden rule of phyllodes tumor, ie, removing 1 inch all around the tumor.” “The nurse practitioner charted that I was NOT interested in continuing to pursue weight loss surgery. This was NOT true. I'm not asked certain questions, and the notes show answers and/or responses that look as if I have provided that information.”
Wrong patient	23 (6.5)	“The Transplant Institute mixed my records with a patient of the same name.” “Plan to change medication based on the lab results of another patient (the wrong patient).” “Inaccurate medical history! Said I was a male with history of binge drinking, family history of gastric cancer, and personal history of malignant hypothermia. All wrong!”
Care plan	15 (4.2)	“The statement that no drain was needed in my hip incision so he sewed me up without one. WOW then I have to wonder what that tube and pouch collecting fluid from my incision was…5 full days of measuring and emptying the suction drain.” “Provider…documented plan of care never discussed with me resulting in delay in my care.”
Sidedness	12 (3.4)	“Wrong eye mentioned for upcoming surgery.” “The wrong breast was indicated on the report making me quite nervous.”
Organization-al process	10 (2.8)	“My notes were sent to the wrong doctor. Someone I no longer see.” “There is a notation that I have 'missed appointments'...Despite me correcting her that the clinic actually cancelled on me multiple times in a row, it is still written up in a way that has me appear noncompliant with treatment.”
Family history	7 (2.0)	“Erroneously stat[ed] that I had no family history of colon cancer; I have a number of colon cancers in my family, including one death.” “Wrong family history—another patient's information in my file.”
Patient demographics	6 (1.7)	“My otherwise fantastic provider inaccurately recorded my sexual orientation identity.” “Reference to my left testicle (I’m female).”
Other	53 (14.9)	
Copy and paste	NA	“Copy and paste an old note missed a lot of current information.”
Billing	NA	“One time it was noted that I had an STD test when I had visited for a cold. I asked my doctor about it and she confirmed that someone had entered the wrong code accidentally. It was quite a shock to see but my doctor resolved it quickly.”
Something important missing	NA	“The note's review of systems was all marked as negative when in fact...I indicated I have chest pain, tightness, palpitations, etc. Also in the [history of present illness] in the same visit, one of the issues we discussed was the profound hypotension I experienced while taking the metoprolol…(SBP ranging 55 at lowest to low 90s). Not a word was mentioned of hypotension in this note. We also talked about the fact that I have had to call in to work frequently due to the ectopic atrial tachycardia HR up to 170s and severe hypotension...none of this is mentioned.”

Abbreviations: BMI, body mass index; HR, heart rate; NA, not available; SBP, systolic blood pressure; STD, sexually transmitted disease.

^a^ Total number of mistakes by type (n = 536) exceeded total number of patients reporting an error (n = 433) because some patients reported more than 1 type of mistake in the comment. Of 433 reports, 77 did not contain enough information to be categorized and were excluded; percentages were therefore calculated using 356 as the denominator.

We examined mistakes that could be associated with the diagnostic process as outlined in the National Academy of Medicine conceptual model.^33^ These mistakes included the following study categories: diagnosis specifically mentioned; medical history; tests, procedures, or results; physical examination; family history; failed communication; wrong patient; and sidedness. Using this broader view, of all patient-reported mistakes, 255 of 433 (58.9%) described at least 1 perceived error that could potentially affect the diagnostic process. A summary of the more common types of patient-reported errors follows.

#### Diagnosis Specifically Mentioned

Patient-reported diagnosis-related mistakes included specific mention of the word *diagnosis* or perceived errors in specific medical diagnoses, including conditions that patients did not have, diagnoses that patients had and thought were relevant but were not recorded, problems or delays in the diagnostic process, or inaccuracy of existing diagnoses (Table 3). Examples included erroneous documentation of diabetes, cancer, and, in 1 instance, HIV infection. One patient wrote, “A history of DCIS [ductal carcinoma in situ] was written in the note as 'disseminated cancer.' I received condolences from another physician.” Another reported a potentially preventable diagnosis of osteoporosis established several years after a missed finding of osteopenia. Several patients reported delayed diagnoses, such as “score added up incorrectly… on depression survey, showing mild depression when really showed severe, mistake found by me, then by psychiatrist reviewing… about a year later.” In some cases, patients noted that diagnoses were made but not communicated to them, such as atrial fibrillation or a “heart attack.”

#### Medical History

Patients frequently described mistakes in their medical history, such as symptoms: “…all marked as negative when in fact... I indicated I have chest pain, tightness, palpitations.” Patients also described situations in which practitioners missed the most important reason for the visit. In 1 example, a pianist reported the physician noted no hand pain when that was the reason for the visit (Table 3). Patients were surprised to find documentation of responses to questions that they perceived were never asked, such as, “My cardiologist repeatedly says that I 'deny' symptoms (such as shortness of breath, etc) that he never asked me about and that I never denied having.”

Some patients noted mistakes in dates or types of operations, including documentation of operations they reported they never had (ie, gall bladder removal, gastric bypass, or hysterectomy). They also reported conflicting information among practitioner notes or between one part of a particular note and another.

#### Medications

In this category, patients described prescription medications that appeared active but that the patient was no longer taking, new prescription medications that the patient was taking but that were missing, and wrong dosages (Table 3). Occasionally, patients reported finding medication not intended for them. There were also many reported errors related to medication allergies, including omission of severe or anaphylactic allergic reactions. Some patients detected discrepancies in medication documentation within the note, with dosages correct in one place but incorrect in another.

#### Tests, Procedures, and Results

Patients identified that some practitioners reported the wrong test result in the note and others who were not aware that more recent results or reports existed. For example, “The provider put the wrong CD4 cell count in my chart. She states 399, however lab results show my CD4 at 219.” Patients also reported mistakes in radiology results or practitioner summaries of radiology reports that made it difficult to determine whether there was clinical improvement or deterioration. In 1 example, different units of measurement resulted in ambiguity: “MRI [magnetic resonance imaging] reads dimensions now 5 × 3 cm compared to prior 5 × 4 mm.” Another commented, “Pathology report summary stated I had 2 positive lymph nodes but [the] detailed report stated 3 positive lymph nodes. That changes the staging and the treatment options. My physician had only read the summary and didn't realize I had 3 positive lymph nodes.” In this case, the patient reportedly notified the physician of the additional positive node, thereby changing the treatment plan because of the wider spread of the cancer. Patients also reported errors of omission, such as a missed lesion in the liver.

#### Other Perceived Errors

Other errors, reported by 53 participants (14.9%), most commonly reflected patients’ perception of something important that was missing from the note (such as active health issues, delivery of vaccines, or recent test results), errors stemming from copy and paste of prior electronic notes, and billing mistakes, such as erroneous codes that implied conditions the patient reportedly did not have. A few patients mentioned errors related to mental health or substance abuse. Several patients reported errors attributable to EHR glitches, such as missing medications after EHR vendor changes, attribution of all tests or vaccination dates to the patient’s birthdate, or a single date for all treatments or operations that reflected the patient’s date of transfer to the organization.

#### Patient Experience With Perceived Mistakes and Attempts to Correct Them

Some patients reported rapid resolution after notifying the practitioner about the perceived error, and several expressed appreciation: “Seeing the notes was very useful ... because it led to not taking an erroneous action... which would otherwise never have been fixed, had the notes not been shared with me.” However, in other instances, patients described experiences of disrespect, such as being derided or ignored (Table 3). One patient commented, “There is a notation that I have 'missed appointments'... despite me correcting her that the clinic actually cancelled on me multiple times in a row, it is still written up in a way that has me appear noncompliant.” Some patients also commented that perceived errors led to emotional or psychological distress, delayed diagnosis or treatment, or lost days at work.

Several patients expressed exhaustion, frustration, and sometimes despair in trying to correct perceived errors: “…repeatedly attempted to fix this, STILL wrong” and “It is almost impossible to change incorrect information [about surgical candidacy]. It took months to do so.” Some were informed that the error could not be corrected or received no response after pointing out perceived errors, such as, “Notes claim … 'BP [blood pressure] in clinic today is 86/56 but she denies any symptoms of lightheadedness or dizziness.' That was inaccurate, I told the nurse who took my vitals I felt faint, lightheaded and weak…I did however call and let the cardiology [department] know about the incorrect report. Unfortunately I don't believe anything was done about it. I never heard back from anyone and the visit note... was never corrected.”

Several patients attributed communication errors, especially those pertaining to events they thought did not occur at the visit, to misunderstanding or misrepresentation; others found the practitioner’s account disingenuous. In a few instances, patients reported seeking a new health care practitioner, especially if their attempts to correct errors were ignored: “This prompted me to find a new provider. Her attitude was that she was not incorrect.”

## Discussion

Reports from more than 22 000 patients who read their practitioners’ notes at 3 US health care centers suggest that patients may play an important role in identifying errors in their records. More than 1 in 5 patients perceived mistakes in their notes, judging more than 40% of them as serious. To our knowledge, no other large-scale, cross-sectional surveys have assessed the frequency of patient-reported errors in ambulatory notes, and these data may help build on findings in smaller pilot programs.^18,28^ Older patients and those with poorer health—patients for whom consequences of errors may be most profound—were approximately twice as likely as younger and healthier patients to identify errors they considered serious, suggesting that note sharing may have particularly important safety implications for those with a heavier burden of illness. In addition, we received reports of mistakes that patients perceived as very serious, suggesting that a feedback mechanism for such findings may be useful for engaging patients in safety.

Despite patients’ rights to review their medical records through the Health Insurance Portability and Accountability Act, systematic checks on the content of notes has been almost absent from clinical documentation. Patient-reported mistakes may help prevent medication errors, diagnostic and treatment delay, and duplicated diagnostic tests and procedures, which occur for approximately 25% to 30% of seriously ill patients.^47^ Patient-reported mistakes in this study surpassed the previously reported error rates of 7% in speech-recognition clinical documents,^48^ suggesting that patients can detect mistakes beyond transcription errors. Lack of routine review of notes by patients may be a missed opportunity not only for EHR accuracy but also for organizational learning.^29,35,49,50,51^

Experts estimate that 12 million Americans experience diagnostic error annually,^52^ and diagnostic errors represent the most frequent category of paid malpractice claims.^33^ Patients who read notes can help ensure that the physician correctly heard and recorded the patient’s symptoms and may also identify mistakes associated with tests and results—known vulnerabilities in some practitioner evaluations.^53,54,55^ Shared notes may also help practitioners to communicate their thought process about a diagnosis.^56^ As safety leaders seek ways to engage patients and families actively in the diagnostic process, sharing clinical notes may be a scalable first step, especially because patient-reported errors related to diagnosis, medical history, physical examination, and tests and results were among the most common patient-reported errors in this study.

Safety experts emphasize learning from patient reports.^57^ However, realizing the potential for shared notes to enhance safety will require broad outreach and education for patients of all ages, races/ethnicities, and educational and health literacy levels. Practitioner support for patient feedback about errors is also important.^58,59^ As reported in this study and elsewhere,^60^ lack of meaningful change after reporting perceived mistakes may be associated with patient frustration and hesitancy to further engage with practitioners. Organizations will need systematic mechanisms for triaging and responding to patient-reported errors, particularly as EHR transparency increases and more patients access their records.

Some patient-reported errors may reflect disagreements between patients and practitioners or may not be errors. However, similar to prior reports,^18,28,40^ the types of very serious mistakes described by patients generally appeared to have relevant clinical implications (Table 3). In addition, in a study^61^ that compared physician notes with audio recordings of unannounced standardized patients, some findings recorded in physician notes did not take place at the visit, similar to patient reports in this study (“Failed communication: did not happen at visit”). Such errors may be associated with use of templated notes if all elements that were not done during the visit (ie, full review of systems, complete physical examination, and time spent counseling) are not accurately edited.

Some patients will identify errors that they themselves do not think are important. Focusing attention instead on errors that they think are most important may be associated with an improved patient experience, even if practitioners believe some of those reports are not errors. However, some concerns that patients do not deem serious may have important health consequences, underscoring the need for further research, patient education, and report triage. As a first step, soliciting patient-reported very serious errors may complement existing error surveillance mechanisms and raise awareness about (at least some) problems that may otherwise go undetected.

### Limitations

This study has limitations. Although similar to other online patient surveys,^62^ the response rate was low at 21.7%. Some patients do not have access to internet or data plans, patient portals, or notes, and results may not reflect the views of patients who do not have access to or do not read their notes online. True patient-reported EHR error rates could therefore be higher or lower than reported here. In addition, although the study involved 3 US health care centers, the patient population overall was predominantly white and educated, characteristics associated with higher likelihood of finding mistakes. Further research with more diverse patient populations is needed.^63^

The data are further limited by self-report and errors unverified by EHR review or patient outcomes, processes beyond our scope. Moreover, patients may have described errors as serious that their practitioners might have categorized differently. However, many of the errors that patients cited were compelling, and the proportion of patients reporting mistakes was similar to reporting tool findings and national surveys of patient-reported errors in EHRs (21% in each).^1,18^ One benefit of patient reports is that they may bring new information about breakdowns in care that are not in the record because they may be missed by practitioners and therefore would not be documented in notes.^29,37,40,64,65^ As a result, not all patient-reported errors can be verified by EHR review. Future research should examine associations between patient-reported errors and safety outcomes, including potential prevention of diagnostic errors.

## Conclusions

The findings suggest that inviting patients to report perceived mistakes in shared visit notes, particularly those that patients believe are very serious, may be associated with improved record accuracy and patient engagement in diagnosis. Developing efficient mechanisms to respond to such reports appears to be important. At a time when patient demand for data is increasing along with federal support for providing patients easy access to health information, shared notes may help enlist patients, families, and practitioners in pursuing greater patient safety collaboratively.

## Notes

Supplement. **eAppendix.** Additional Information on Open Notes History and Note-Reading Access and Experience **eTable.** Patient-Reported Error Categories, Adapted From Patient-Reported Errors in Visit Notes Using an Online Reporting Tool **eReferences**
