# Supplementary material for: Frequency and Types of Patient-Reported Errors in Electronic Health Record Ambulatory Care Notes
Source: JAMA Netw Open. 2020 Jun 9;3(6):e205867. doi: 10.1001/jamanetworkopen.2020.5867 (PMC7284300; doi:10.1001/jamanetworkopen.2020.5867)
Supplement: Supplement. — eAppendix. Additional Information on Open Notes History and Note-Reading Access and Experience eTable. Patient-Reported Error Categories, Adapted From Patient-Reported Errors in Visit Notes Using an Online Reporting Tool eReferences [file jamanetwopen-3-e205867-s001.pdf]

Supplementary Online Content

Bell SK, Delbanco T, Elmore JG, et al. Frequency and types of patient-reported errors in electronic health record ambulatory care notes. *JAMA Netw Open*. 2020;3(6):e205867. doi:10.1001/jamanetworkopen.2020.5867

eAppendix. Additional Information on Open Notes History and Note-Reading Access and Experience
eTable. Patient-Reported Error Categories, Adapted From Patient-Reported Errors in Visit Notes Using an Online Reporting Tool
eReferences

This supplementary material has been provided by the authors to give readers additional information about their work.

© 2020 Bell SK et al. *JAMA Network Open.*

eAppendix. History of Open Notes

Open Notes began as a research and demonstration study in 2010, with 105 primary care physicians inviting 20,000 of their patients to read their visit notes online through the patient portal at 3 healthcare organizations: BIDMC, Geisinger, and UW, the same organizations participating in this follow up study. Results of the initial study were published in 2012, demonstrating several patient-reported benefits and little negative impact on physicians.[1] At the end of the trial year, 99% of patients wanted open notes to continue; none of the physicians discontinued note access.[1] Today, over 44 million patients across the U.S. have access to their notes through the patient portal, and at least 10 other countries are also sharing transparent notes with patients.[2,3]

Although Open Notes began with primary care notes, by 2014 the practice of sharing notes at the 3 study organizations had spread to virtually every medical and surgical ambulatory clinic and to all healthcare professionals who sign clinical notes (doctors, nurses, nurse practitioners, physician assistants, dieticians, etc). Today, notes have been shared and studied in various clinical settings including pediatrics, oncology, obstetrics and gynecology, dermatology (including pathology reports), mental health, and in-patient care.[4–9]

Access to notes and note-reading experience among the survey respondents

BIDMC used a homegrown EHR and patient portal ("PatientSite"). Geisinger and UW used Epic as the vendor for their EHRs and patient portals, however their portals were stylized to each organization's preferences (i.e. "MyGeisinger" and "eCare," respectively). Portal registration at each organization was at the discretion of individual practices. In many, front desk personnel offered portal registration. Patients accessed notes on the portal similar to accessing medications, problem lists, or results, although this process was not the same on each patient portal. Although in some patient portals access to notes requires several "clicks," and despite differences in portal design and in patient education across sites, the majority (71.4%) of patients reported that accessing notes was very easy (8-10 on a scale of 0-10).[10] Similarly, when (n=763) nonreaders were asked why they did not read notes, about half reported that they forgot or did not know that visit notes were available. Of all nonreaders, 6% reported that they looked on the portal but were not able to find the notes.[10]

The survey was extensively tested and revised with outside reviewers, focus groups, and psychometric testing of some items.[10] Patients at BIDMC and UW received automated messages notifying them of the availability of a new visit note; those at Geisinger did not.[10]

The patient survey was administered online through REDCap. Each invitation held a unique identifier that could not be used again. We tracked eligible patients who were not invited and differentiated between respondents and nonrespondents, and tracked withdrawal of consent as previously reported.[10]

© 2020 Bell SK et al. *JAMA Network Open.*

eTable. Patient-reported error categories, adapted from patient-reported errors in visit notes using an online reporting tool (Bell et al BMJ Qual Saf 2017)

Medical history

- Description of the clinical course
- Symptoms: what brought patient to the doctor, and/or what makes symptoms better or worse
- Prior medical problems or conditions
- Dates or types of surgeries

"Diagnosis" mentioned

- Any patient comment that uses the words diagnosis or misdiagnosis/misdiagnosed
- Misidentification of patient diagnoses; ie specific conditions listed or omitted in error
- Delay in reaching diagnosis
- Failure to communicate the diagnosis to the patient

Medications/allergies

- Wrong medication: wrong dose, no longer taking, taking but missing from list, prescription errors
- Medication allergy or reaction not listed, or listed incorrectly

Social History

- Alcohol use, smoking, drug use
- Patient's job/employment, place of residence
- Family members names, ages, place of residence

Family history

- Inaccurate or missing family medical conditions: especially history of cancer, diabetes, other heritable diseases

Physical exam

- Description of physical exam
- Documentation of exam or parts of exam that patient perceives were not done

Labs/tests/procedures/results

- Test results or communication about them
- Delay in testing, such as lack of necessary paperwork to obtain recommended tests

Did not happen in visit: Failed communication

- Note indicates that patient declined a treatment, test, or surgery, but patient disagrees
- Documentation of counseling or other discussion that patient perceives did not occur

Care plan:

- Next steps or treatment
- Problematic coordination of care with other providers
- Plan for referrals or follow up visits

Sidedness:

- Description of "right" vs "left"

Patient demographics

- Patient name, age, gender, race, ethnicity, or other describing characteristics

Wrong patient

- Note entered on the wrong patient

Doctor or organization-related

- Name, location, time of appointment
- Long wait, service issues

© 2020 Bell SK et al. *JAMA Network Open.*

- Wrong name of doctor, or wrong doctor receiving results/referral notes

Not enough information/Not applicable

- Category can not be determined from limited description

Other:

- Doesn’t fit into any of the above

eReferences

1. Delbanco T, Walker J, Bell SK, et al. Inviting patients to read their doctors' notes: A quasi-experimental study and a look ahead. *Ann Intern Med*. 2012;157(7):461-470. doi:10.7326/0003-4819-157-7-201210020-00002

2. Hägglund M, Desroches C, Petersen C, Scandurra I. Patients' access to health records. *BMJ*. 2019;367(October):2-3. doi:10.1136/bmj.l5725

3. OpenNotes®. OpenNotes Map. https://www.opennotes.org/join/map/.

4. Bell SK, Folcarelli P, Fossa A, et al. Tackling Ambulatory Safety Risks Through Patient Engagement: What 10,000 Patients and Families Say About Safety-Related Knowledge, Behaviors, and Attitudes After Reading Visit Notes. *J Patient Saf*. 2018;00(00):1-9. doi:10.1097/PTS.0000000000000494

5. Herlihy M, Harcourt K, Fossa A, Folcarelli PH, Golen T, Bell S. An Opportunity to Engage Obstetric and Gynecology Patients in Safety and Quality through Shared Visit Notes. *Obstet Gynecol*. 2019;133(1):S1-3. doi:10.1097/01.aog.0000559076.53573.ab

6. Shucard H, Piepkorn MW, Reisch LM, et al. Dermatopathologists' Experience With and Perceptions of Patient Online Access to Pathologic Test Result Reports. 2020;90024:1-5. doi:10.1001/jamadermatol.2019.4194

7. Dobscha SK, Denneson LM, Jacobson LE, Williams HB, Cromer R, Woods S. VA mental health clinician experiences and attitudes toward OpenNotes. *Gen Hosp Psychiatry*. 2016;38:89-93. doi:10.1016/j.genhosppsych.2015.08.001

8. O'Neill S, Chimowitz H, Leveille S, Walker J. Embracing the new age of transparency: mental health patients reading their psychotherapy notes online. *J Ment Heal*. 2019;28(5):1-9. doi:10.1080/09638237.2019.1644490

9. Weinert C. Giving Doctors' Daily Progress Notes to Hospitalized Patients and Families to Improve Patient Experience. *Am J Med Qual*. 2015;32(1):58-65. doi:10.1177/1062860615610424

10. Walker J, Leveille S, Bell S, et al. OpenNotes After 7 Years: Patient Experiences With Ongoing Access to Their Clinicians' Outpatient Visit Notes. *JMIR*. 2019;21(5):e13876. doi:10.2196/13876

© 2020 Bell SK et al. *JAMA Network Open.*
